# The relationship between dietary intakes and plasma concentrations of PUFA in school-age children from the Avon Longitudinal Study of Parents and Children (ALSPAC) cohort

**DOI:** 10.1017/S0007114521002191

**Published:** 2022-05-14

**Authors:** Genevieve Buckland, Sandra de Silva Johnson, Laura Johnson, Caroline M. Taylor, Louise R. Jones, Pauline M. Emmett

**Affiliations:** 1 Centre for Academic Child Health, Bristol Medical School, University of Bristol, Bristol BS8 1NU, UK; 2 Centre for Exercise, Nutrition and Health Sciences, School for Policy Studies, University of Bristol, Bristol, UK

**Keywords:** PUFA, Avon Longitudinal Study of Parents and Children (ALSPAC), Paediatric, Biomarker

## Abstract

An adequate intake of PUFA plays a vital role in human health. Therefore, it is important to assess PUFA intakes in different populations and validate them with biomarkers, but only a few small studies are in paediatric populations. We calculated the dietary intake of PUFA and their main food sources in children and assessed associations between PUFA intakes and plasma proportions. Dietary intakes of 7-year-old children (*n* 8242) enrolled in the Avon Longitudinal Study of Parents and Children were calculated from the parental-completed FFQ. Plasma PUFA were measured in 5571 children 8 months later, and 4380 children had complete dietary and plasma data. The association between dietary and plasma PUFA proportions was estimated using Spearman’s correlation coefficients, quintile cross-classification and Cohen’s *κ* coefficients. Mean total PUFA intake was 13·2 g/d (sd 4·2), contributing 6·5 % of total energy intake; *n*-6 PUFA contributed 5·2 % and *n*-3 PUFA 0·7 %. The *n*-6:*n*-3 ratio was 7·9:1. Mean intakes of EPA and DHA were 35·7 mg/d and 49·7 mg/d, respectively. Most *n*-3 and *n*-6 PUFA intakes were weakly correlated with their respective plasma lipids (0·07 ≤ *r* ≤ 0·16, *P* < 0·001). The correlation between dietary and plasma DHA was stronger though (*r* = 0·34, *P* < 0·001), supported by a modest level of agreement between quintiles (*k* = 0·32). The results indicate that the FFQ was able to reasonably rank the long-chain (LC) PUFA, DHA, in this paediatric population. Public health initiatives need to address the suboptimal ratio of *n*-6:*n*-3 PUFA and very low *n*-3 LC-PUFA intakes in school-age children in the UK.

PUFA are essential for human growth and development, forming a crucial part in membrane structures and brain and retinal development during infancy^(1)^. They may also play an important role in modulating the risk of CVD, inflammatory and neurodegenerative diseases^(2,3,4)^. PUFA consist of two distinct families: *n*-3 and *n*-6. The medium-chain parent fatty acids (FA), *n*-3 *α*-linolenic acid (ALA) and *n*-6 linoleic acid (LA) are termed essential because they cannot be synthesised endogenously and so need to be provided by diet. In contrast, the *n*-3 and *n*-6 long-chain (LC) PUFA can be derived either from the diet or endogenously synthesised from the parent PUFA. The *n*-3 and *n*-6 PUFA have distinct physiological functions^(1,4,5)^. A low ratio of *n*-6:*n*-3 PUFA in the diet is important for health, since high ratios favour a pro-inflammatory state^(3,4)^. Modern Western diets are generally low in *n*-3 PUFA, particularly in the marine LC-PUFA (EPA and DHA) while high in *n*-6 PUFA, resulting in an *n*-6:*n*-3 ratio often reaching up to 15–16:1^(6)^. Therefore, lowering the current ratio is recommended, since an *n*-6:*n*-3 ratio of 2–3:1 is associated with reduced risk of many chronic inflammatory-related diseases^(4)^. A high ratio of *n*-6:*n*-3 PUFA and/or inadequate EPA and DHA early in life may also be a potential risk factor for a range of neurodevelopmental cognitive disorders in childhood^(7)^.

Many countries, including the UK, have made public health recommendations to replace the consumption of SFA with PUFA^(8,9,10,11)^. The UK Scientific Advisory Committee on Nutrition recommends that 6·5 % of total energy (TE) intake should be from PUFA^(10)^. The European Food Safety Authority recommends an intake of 250 mg/d of EPA and DHA^(12)^. However, many Western populations fall well below this intake^(13,14,15,16,17,18)^. Data from the nationally representative UK National Diet and Nutrition Survey (NDNS) showed that while total and *n*-6 PUFA intakes were in line with dietary guidelines, most children failed to meet recommended minimum weekly fish intakes^(19)^. However, direct measures of EPA and DHA were not available. It is particularly relevant to assess adequacy of PUFA intakes in paediatric populations as suboptimal PUFA intakes early in life may modulate disease risk throughout the life course^(7,20)^. It is also essential to validate the tools used to assess dietary PUFA intakes, which is generally done by studying PUFA concentrations in blood and tissue^(21)^. Numerous biomarker validation studies in adults have compared PUFA intakes estimated using dietary questionnaires, records or recalls with tissue biomarkers, including FA in plasma, phospholipids, erythrocyte membranes and platelets or in adipose tissue^(22,23,24,25,26)^. However, validation studies conducted in children are limited and mostly based on small sample sizes (*n* 35–404)^(27,28,29,30)^. Estimating dietary intake is particularly challenging in children, and reporting error (notably under-reporting) can vary by age group^(31,32)^.

Therefore, the objectives of this study were to (1) assess the dietary intake and food sources of *n*-3 and *n*-6 PUFA within a paediatric population from the UK (*n* 8242) and (2) measure the correlations between PUFA intakes estimated through FFQ and PUFA concentrations in plasma (*n* 4380) in children from the Avon Longitudinal Study of Parents and Children (ALSPAC).

## Methods

### Study cohort and participants

**Fig. 1.**
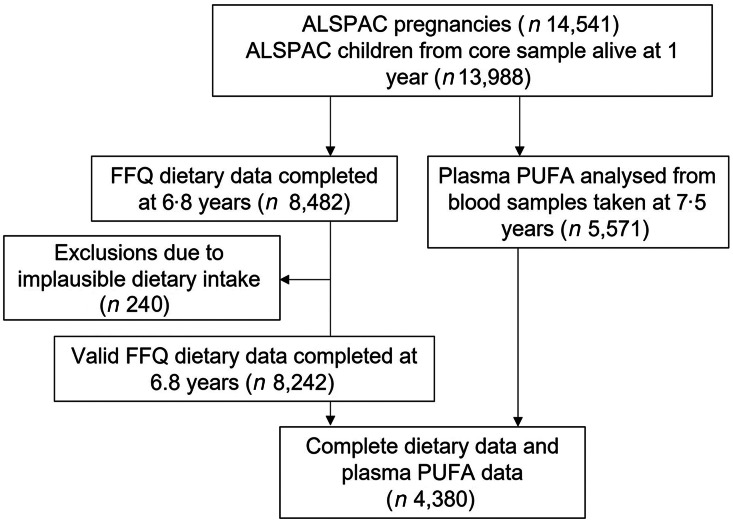
Study flow diagram for participant data from the Avon Longitudinal Study of Parents and Children (ALSPAC).

The study participants were the core index children (first generation = G1) from ALSPAC, a transgenerational prospective birth cohort established to investigate the determinants of health and disease across the life course, including childhood development and growth. Full details of the cohort and study design have been described previously^(33,34,35)^ and are also available on the ALSPAC website (www.alspac.bris.ac.uk). In addition, the study website contains details of all the data that is available through a fully searchable data dictionary and variable search tool (http://www.bristol.ac.uk/alspac/researchers/our-data/). In 1991–1992, 14 541 eligible pregnant women from the Southwest of England were enrolled into the study, resulting in 13 988 children alive at 1 year and followed since birth. During follow-up, extensive data have been regularly collected on the parents and their children, primarily using questionnaires, medical records, biological samples and clinical visits. The current study uses data from the child cohort when aged 6·8 (sd 0·1) years whose parents completed a child-based FFQ in 1997–1999 (*n* 8482) and from the children who took part in a research clinic at age 7·5 (sd 0·2) years and had blood samples collected and analysed (*n* 4380 children had blood samples and FFQ data), see Fig. 1 for study flow diagram. Ethics approval for the study was obtained from the ALSPAC Ethics and Law Committee and the Local Research Ethics Committee (http://www.bristol.ac.uk/alspac/researchers/research-ethics/) and conformed to the Declaration of Helsinki. Consent for biological samples was collected in accordance with the Human Tissue Act.

### Dietary data

The parental-completed FFQ was adapted from the original FFQ used to assess maternal diet in ALSPAC at 32 weeks of pregnancy^(36)^, with full details published previously^(37)^. In summary, the questionnaire contained a series of questions enquiring about the frequency of the child’s habitual consumption of eighty different food and drinks and included questions about school meals and food items often consumed by children. The frequency ranges used were ‘never or rarely’, ‘once every 2 weeks’, ‘1–3 times a week’, ‘4–7 times a week’ and ‘more than once a day’. There were five questions directly relating to fish and seafood intake. These foods are high in *n*-3 LC-PUFA and thus allowed an estimate of *n*-3 LC-PUFA intakes particularly from fish sources. Foods normally consumed every day and in a variety of forms, such as bread, milk and fat spreads, were questioned in more detail. Standard portion sizes^(38)^ for children in this age group were used in combination with the reported frequency of consumption of each food/drink to calculate dietary intakes. Energy and nutrients intakes were estimated using the nutrient content of foods based on 5th edition of McCance and Widdowson’s (M&W) food tables^(39)^. The food items and portion sizes assessed for the school meal section of the FFQ were informed by school menus collected at the time from local schools.

### Estimation of PUFA intake

A food composition database was created in order to calculate the children’s intake of total, *n*-3 and *n*-6 PUFA and individual PUFA (LA, ALA, arachidonic acid (AA), EPA and DHA). The PUFA composition of food items covered in the 7-year FFQ was primarily determined using the electronic version of M&W food composition tables (6th edition, 2002)^(40)^. When necessary, this was supplemented with the M&W Fatty Acids Supplement (Ministry of Agriculture Fisheries and Food (MAFF), 1998) and data from the NDNS database^(41)^. A manual matching process was employed to combine ALSPAC food codes with appropriate M&W code. If no exact match was found, a similar food item close to the original was used, resulting in all foods in the FFQ with any fat content (332 food items) being covered in the food composition database.

### Plasma fatty acids

Plasma obtained from the non-fasting blood samples was stored at –70°C, thawed once to obtain a 100 µl aliquot that was refrozen and shipped by airfreight to Rockville, MD, USA, and then thawed for final analyses^(42)^. Plasma FA were extracted using transmethylation of lipids with acetyl chloride and methanol^(43,44)^. Chromatographic separation of the FA methyl esters was achieved via a fast gas chromatography 6890 Plus LAN system (Agilent Technologies) coupled with a fused-silica, narrow bored DB-FFAP capillary column (Agilent 127–32H2, 15m × 0·1 mm I.D. × 0·1 mm film thickness). Assays were carried out during 2009–2010 with the measurement of twenty-two FA, eleven of which were PUFA.

### Statistical analysis

Analyses were performed using SPSS (version 19) and STATA 15 (Statacorp.). A total of 240 (2·8 %) of the original 8482 participants with FFQ data were excluded from the statistical analysis due to implausible dietary data, using cut-offs <15 000 and >140 000 kJ/week, based on inspecting the histogram of weekly energy intake. This gave a final study sample of 8242 participants with valid FFQ data and 4380 participants with both valid FFQ and blood plasma FA data. The analyses were carried in all participants and stratified by sex. The dietary and plasma PUFA data were assessed for normality, and since the majority of the data was not normally distributed, non-parametric tests were used (the data were not transformed). The children’s daily PUFA intake was summarised as medians and interquartile ranges, and as a percentage of TE intake. Plasma PUFA concentrations were presented as percentage of total FA. The contribution of dietary *n*-3 and *n*-6 PUFA from eleven food groups was calculated and expressed as median daily intake and percentages of total PUFA intake (calculated at an individual level). These food groups encompassed all the individual food items (except soft drinks) covered in the FFQ and consisted of (1) vegetables, pulses and potatoes; (2) bread, cereals and bakery products; (3) meat and meat products; (4) fish and fish products; (5) milk and milk products; (6) fat spreads and cooking fat; (7) crisps and savoury snacks; (8) nuts and seeds; (9) egg and egg dishes; (10) fruit and (11) sugar, preserves and confectionary. The contribution of dietary DHA and EPA (mg/d) from different categories of fish and seafood was also calculated.

The correlation between crude and energy-adjusted dietary PUFA intakes and plasma PUFA proportions was assessed by Spearman’s correlation coefficients (*r*). PUFA intakes were not log-transformed but were energy adjusted using the energy density method, by dividing each individual’s PUFA intake by their TE intake and then multiplying by 7000 (approximately the median energy intake in kJ/d)^(45)^. Cross-classification analysis was used to evaluate the agreement between the two PUFA measures. Energy-adjusted dietary PUFA intakes were classified into quintiles and then cross-tabulated with quintiles of the respective PUFA plasma proportion. Discordance and agreement in quintile rankings were evaluated by calculating the percentage of participants classified in the same quintile, same or adjacent quintile and opposite quintile. In addition, Cohen’s weighted kappa statistics (*κ*w) and 95 % CI were calculated for quintiles of energy-adjusted PUFA intakes and plasma PUFA proportions, since they consider agreements that were due to chance. The strength of the correlations (*r*) and agreements (*κ*w) was evaluated as poor (<0·2), moderate (0·2–0·59) or good (>0·6)^(46)^.

## Results

The characteristics of the 8242, seven-year-old children with FFQ data and 4380 children with both FFQ and plasma FA data are outlined in Table 1. In the sample of 8242 children, there was a mean energy intake of 7687 (sd 1859) kJ/d. Fat intake (75·7 g/d) contributed 37·1 % to TE intake, of which 14·7 % of energy was from SFA, 11·8 % from MUFA and 6·5 % from PUFA (13·2 g/d). The sub-sample with both dietary and plasma FA data had a lower daily energy intake, mothers with a higher education, a higher family social class and were less overweight/obese compared with the sample with only FFQ data.

**Table 1.** Characteristics and daily nutrient intakes of the 8242, seven-year-old children from Avon Longitudinal Study of Parents and Children (ALSPAC) with dietary data compared with the 4380 with both plasma and dietary data (Numbers and percentages; mean values and standard deviation)

Characteristics	Sample with FFQ data (*n* 8242)		Sample with FFQ and plasma fatty acid data (*n* 4380)		*P*-value^*^
	*n*	%	*n*	%	
Sex, male	4225	51·0	2266	52·0	0·360
BMI, overweight/obese (kg/m^2^)	1035	16·1	650	14·9	<0·001
Maternal educational status					
Low status (none, CSE, vocational)	1902	23·7	851	19·7	
Medium status (O-Level)	2841	35·4	1531	35·2	
High status (A-level and degree)	3277	40·9	1941	45·0	<0·001
Highest household social class					
Grade I and II (highest)	2203	28·9	1262	30·40	
Grade III (manual and non-manual)	4126	54·0	2215	53·4	
Grade IV and V (lowest)	1307	17·1	675	16·3	0·002
Total energy, kJ/d					
Mean	7687		7627		0·002
sd	1859		1763		
Carbohydrate intake					
g/d					
Mean	238·9		237·1		0·003
sd	59·8		56·5		
% energy					
Mean	51·9		52·0		0·487
sd	3·8		3·8		
Protein intake					
g/d					
Mean	65·1		64·8		0·053
sd	16·4		15·8		
% energy					
Mean	14·2		14·2		0·163
sd	1·8		1·8		
Total fat intake					
g/d					
Mean	75·7		75·1		0·001
sd	20·3		19·4		
% energy					
Mean	37·1		37·0		0·234
sd	3·5		3·5		
SFA					
g/d					
Mean	30·1		29·8		0·002
sd	9·2		8·8		
% energy					
Mean	14·7		14·7		0·307
sd	2·5		2·4		
MUFA					
g/d					
Mean	24·2		23·9		<0·001
sd	6·5		6·2		
% energy					
Mean	11·8		11·8		0·008
sd	1·2		1·2		
PUFA					
g/d					
Mean	13·2		13·1		0·148
sd	4·2		4·0		
% energy					
Mean	6·5		6·5		0·195
sd	1·4		1·4		

CSE, certificate of secondary education.

* *P*-value comparing difference between sample with both FFQ and plasma FA data and sample with only FFQ data (*χ* ^2^ for categorical variables and *t*-test for continuous variables).

### Dietary and plasma PUFA

The reported intake of dietary FA and proportions of plasma FA (calculated as percentage of total FA) is shown in Table 2, along with data on the PUFA subtypes. The majority of PUFA were consumed in the form of *n*-6 PUFA; 80·3 % of total PUFA and 5·2 % of TE. This was mainly due to intake of LA, which contributed to a mean of 5·1 % of TE. *n*-3 PUFA accounted for 10·6 % of the total PUFA (0·7 % of TE), with the majority in the form of ALA. The daily intake of the DHA was 49·7 mg/d with 10 % of children having <15 mg/d. The LC *n*-3 PUFA (DHA and EPA) average intake was 85·4 mg/d. The *n*-6:*n*-3 ratio in the diet was 7·9:1.

**Table 2.** Daily dietary intakes of fatty acids estimated from a FFQ and plasma fatty acid proportions in 7-year old children from Avon Longitudinal Study of Parents and Children (ALSPAC) (Mean values and standard deviation; median and interquartile range)

Fatty acids (total and subtypes)	Mean	sd	Median	IQR	Mean (% of energy)	sd
Dietary intake (*n* 8242)						
Total fatty acids, g/d	75·7	20·3	73·9	62·2–87·4	37·1	3·5
SFA, g/d	30·1	9·2	29·0	23·8–35·3	14·7	2·5
MUFA, g/d	24·2	6·5	23·5	19·7–27·9	11·8	1·2
PUFA, g/d	13·2	4·2	12·8	10·3–15·8	6·5	1·4
*n*-6 PUFA, g/d	10·6	3·5	10·3	8·2–12·8	5·2	1·2
18 : 2*n*-6 (LA), g/d	10·30	3·4	10·0	7·9–12·4	5·1	1·2
20 : 4*n*-6 (AA), g/d	0·05	0·02	0·05	0·04–0·06	0·02	0·02
*n*-3 PUFA, g/d	1·4	0·4	1·3	1·1–1·7	0·7	0·1
18 : 3*n*-3 (ALA), g/d	1·3	0·4	1·2	1·0–1·5	0·6	0·1
22 : 6*n*-3 (DHA – total), mg/d	49·7	44·8	38·1	23·0–60·9	0·025	0·02
22 : 6*n*-3 (DHA – from fish only), mg/d	35·0	42·2	20·5	11·9–43·1	0·017	0·02
20 : 5*n*-3 (EPA – total), mg/d	35·7	26·6	29·2	20·6–42·5	0·018	0·01
20 : 5*n*-3 (EPA – from fish only), mg/d	19·5	24·5	12·2	6·5–24·4	0·010	0·01
LC *n*-3 PUFA (EPA + DHA), mg/d	85·4	70·4	66·5	45·2–102·7	0·042	0·03
Total *n*-6/Total *n*-3	7·9	2·3	7·4	6·3–9·0	7·9	2·3
Plasma proportion, % of total fatty acids (*n* 4380)						
SFA	29·3	3·2	29·6	27·4–31·5	–	
MUFA	26·9	3·2	26·7	24·8–28·8	–	
*n*-6 PUFA	39·8	3·9	39·9	37·3–42·4	–	
*n*-3 PUFA	3·9	0·8	3·8	3·4–4·3	–	
18 : 2*n*-6 (LA)	30·6	3·2	30·7	28·6–32·7	–	
20 : 4*n*-6 (AA)	6·4	1·3	6·4	5·5–7·3	–	
18 : 3*n*-3 (ALA)	0·7	0·3	0·7	0·5–0·8	–	
22 : 6*n*-3 (DHA)	1·9	0·5	1·8	1·5–2·3	–	
20 : 5*n*-3 (EPA)	0·6	0·2	0·6	0·5–0·7	–	

QR, quartile range (25th percentile–75th percentile); AA, arachidonic acid; ALA, *α*-linolenic acid; LC, long-chain.

The median concentration of total FA in plasma was 2·26 mg/ml (1·9–2·6 mg/ml for 25th and 75th percentile range). The PUFA plasma proportions were dominated by *n*-6 PUFA, particularly LA (30·6 % of total plasma FA). AA (a LC *n*-6 PUFA) contributed 6·4 % of total plasma FA, whereas *n*-3 PUFA (ALA, DHA and EPA) contributed only 3·2 % of total FA and the contribution of DHA was more than twice that of either ALA or EPA. The PUFA intakes and PUFA plasma proportions are presented separately for females and males in Supplementary Tables 1 and 2, respectively. Statistical comparison of PUFA intakes and plasma proportions by sex indicated differences unlikely to be explained by chance; however, in absolute terms, the differences were minimal.

### Dietary sources of PUFA intake

The mean daily intakes and percentage contribution to *n*-6 and *n*-3 PUFA and DHA intakes according to food groups are shown in Table 3 (online Supplementary Table 3 for sex-specific intakes). The highest intake of *n*-6 PUFA was from cereal-based products and from fat spreads and cooking fat, together contributing to almost half of *n*-6 PUFA intake. Further important sources were fats used in vegetable and potato dishes and in meat and meat products. The main source of *n*-3 PUFA was vegetable fat used in vegetable and potato dishes (28·5 %), followed by cereal products, meat and meat products and milk and milk products. The major dietary source of DHA and EPA was fish (contributing to 59·2 % of DHA and 45·9 % of EPA intake). Other dietary sources of the LC-PUFA in these children were meat and meat products, eggs (for DHA), and fats and spreads and milk and milk products (for EPA). Most other food groups provided no DHA or EPA. In terms of the different types of fish and seafood, coated fish contributed most to the children’s DHA and EPA intake, providing a mean of 7·9 mg/d and 6·8 mg/d, respectively (Table 4). Another major source of LC-PUFA was from oily fish and tuna (canned or fresh). School meals contributed to 10·5 % and 9·5 % of DHA and EPA from fish, respectively. Out of the cohort of 8242 children, 568 (6·9 %) did not consume any fish or seafood.

**Table 3.** Daily intake and percentage contribution of total *n*-6, total *n*-3 PUFA, DHA and EPA intakes by food group estimated from a parental-completed FFQ when the child was aged 7 years (*n* 8242)

	n-6 PUFA intake total				n-3 PUFA intake total				Long-chain PUFA intake							
									DHA				EPA			
	g/day		% daily total n-6		g/day		daily % total n-3		mg/day		% daily total DHA		mg/day		% daily total EPA	
	Median	IQR	Mean	sd	Median	IQR	Mean	sd	Median	IQR	Mean	sd	Median	IQR	Mean	sd
Vegetables and potatoes	1·45	1·0–2·2	16·5	8·5	0·37	0·3–0·5	28·5	8·8	0·00	0·0–0·0	0·0		0·32	0·2–0·3	1·2	1·8
Cereal and cereal products	2·31	1·7–3·0	23·5	8·2	0·27	0·2–0·4	20·7	6·9	0·11	0·0–0·3	1·9	7·8	0·56	0·4–0·7	2·5	4·0
Meat and meat products	1·61	1·2–2·2	16·7	7·6	0·24	0·2–0·3	18·7	7·6	6·52	5·4–8·5	21·2	17·6	2·77	2·1–3·9	11·1	8·9
Fish and fish dishes	0·58	0·2–0·6	4·4	3·2	0·06	0·0–0·1	5·8	5·7	20·50	11·9–43·1	59·2	24·4	12·23	6·5–24·4	45·9	23·0
Milk and milk products	0·39	0·3–0·5	4·3	2·4	0·15	0·1–0·2	12·5	5·9	0·00	0·0–0·0	0·0	0·0	3·30	3·2–8·8	14·7	13·0
Fat and spreads	2·47	0·6–3·7	22·4	15·9	0·08	0·0–0·2	6·9	6·6	0·00	0·0–0·4	0·9	4·2	6·93	1·4–11·6	21·4	18·3
Crisps and savoury snacks	0·32	0·3–0·9	5·9	4·2	0·03	0·0–0·1	4·2	3·0	0·00	0·0–0·0	0·0	0·0	0·00	0·0–0·0	0·0	0·0
Nuts and seeds	0·00	0·0–2·2	2·8	5·7	0·00	0·0–0·0	0·3	0·7	0·00	0·0–0·0	0·0	0·0	0·00	0·0–0·0	0·0	0·0
Egg and egg dishes	0·08	0·0–0·3	1·8	2·0	0·01	0·0–0·0	1·0	1·1	3·74	1·1–12·2	16·6	17·7	0·44	0·0–1·7	3·1	4·1
Fruit	0·00	0·0–0·0	0·1	0·1	0·00	0·0–0·1	0·7	1·0	0·00	0·0–0·0	0·0	0·0	0·00	0·0–0·0	0·0	0·0
Sugar, preserves and confectionary	0·10	0·1–0·2	1·6	1·7	0·00	0·0–0·1	0·4	0·4	0·00	0·0–0·0	0·0	0·0	0·00	0·0–0·0	0·0	0·0
Total	10·3	8·2–12·8	100		1·33	1·1–1·7	100		38·10	23·0–60·9	100		29·2	20·6–42·5	100	

Abbreviations: IQR, Inter-quartile range (25th percentile–75th percentile); n-6, omega-6 series; PUFA, Polyunsaturated fatty acids; n-3, omega-3 series; DHA, Docosahexaenoic acid; EPA, Eicosapentaenoic acid.

**Table 4.** Contribution of different types of fish to DHA intake estimated from a parental-completed FFQ when the child was aged 7 years (*n* 8242)

	DHA				EPA			
	mg/day		% from fish		mg/day		% from fish	
Type of fish consumed	Mean	sd	Mean	sd	Mean	sd	Mean	sd
Shellfish	0·27	0·9	1·0	4·9	0·40	1·4	2·1	7·5
Coated fish	7·85	5·9	40·6	34·2	6·80	1·5	51·9	33·7
White fish	5·24	10·0	12·4	20.1	2·65	5·0	11·6	18·8
Tuna (tinned and fresh)	8·31	12·3	22·4	27·6	1·43	2·1	10·7	18·1
Oily/fatty fish	11·32	31·1	13·1	26·1	7·30	20·2	14·2	28·0
School meal fish	2·87	4·4	10·5	20·1	1·00	1·5	9·5	18·9
Total FA from fish	35·00	42·2	100		19·50	24·5	100	

Abbreviations: DHA, docosahexaenoic acid; EPA, eicosapentaenoic acid.

### Validation analyses

The correlation between energy-adjusted dietary PUFA intakes and PUFA plasma proportions is presented in Table 5 (there were minimal differences in the correlations using crude and energy-adjusted PUFA intakes, so only energy-adjusted results are presented). Overall, the dietary intakes of the parent *n*-6 and *n*-3 FA (LA and ALA) were weakly correlated with their respective plasma lipid concentrations (*r* = 0·16, *P* < 0·001 and *r* = 0·14, *P* < 0·001, respectively). There were also weak correlations between dietary and plasma AA and between dietary and plasma EPA (*r* = 0·08, *P* < 0·001 and *r* = 0·10, *P* < 0·001, respectively). The strongest correlation in our study was between dietary and plasma DHA (*r* = 0·34, *P* < 0·001), explaining about 8 % of the variance. The correlations were similar when female and male participants were analysed separately (online Supplementary Tables 5 and 6, respectively). With regard to correlations between different types of PUFA, the precursor of the *n*-6 series, dietary LA, was not correlated with plasma AA but it was weakly negatively correlated with plasma concentrations of EPA. For the *n*-3 PUFA, there were significant but weak positive correlations between dietary ALA, the precursor of the *n*-3 series, and EPA and DHA, and between dietary EPA and plasma DHA and vice versa.

**Table 5.** Spearman’s correlation coefficients (*r*) between plasma concentrations and energy-adjusted dietary intakes of *n*-3 and *n*-6 PUFA (*n* 4380)

Dietary PUFA	Plasma PUFA									
	*n*-6 FA				*n*-3 FA					
	18:2 (LA)	*P*-value	20:4 (AA)	*P*-value	18:3 (ALA)	*P*-value	20:5 (EPA)	*P*-value	22:6 (DHA)	*P*-value
Total PUFA	0·163	<0·001	0·013	0·388	0·003	0·830	-0·140	<0·001	0·011	0·488
Total *n*-6	0·161	<0·001	0·012	0·448	-0·001	0·956	-0·149	<0·001	-0·022	0·145
18 : 2*n*-6 (LA)	**0·162**	<0·001	0·011	0·465	-0·004	0·813	-0·151	<0·001	-0·024	0·117
20 : 4*n*-6 (AA)	-0·056	<0·001	**0·079**	<0·001	0·077	<0·001	0·149	<0·001	0·197	<0·001
Total *n*-3	0·003	0·862	0·023	0·137	0·138	<0·001	0·114	<0·001	0·170	<0·001
18 : 3*n*-3 (ALA)	0·010	0·491	0·003	0·843	**0·138**	<0·001	0·086	<0·001	0·113	<0·001
20 : 5*n*-3 (EPA)	0·046	0·002	0·031	0·043	0·018	0·223	**0·102**	<0·001	0·266	<0·001
22 : 6*n*-3 (DHA)	0·030	0·044	0·057	<0·001	0·052	<0·001	0·123	<0·001	**0·341**	<0·001

FA, fatty acids; LA, linoleic acid; AA, arachidonic acid; ALA, *α*-linolenic acid.

Values in bold indicate correlation coefficient between dietary PUFA and corresponding PUFA in plasma.

Cross-classification of quintiles of dietary and plasma PUFA subtypes showed that 54–79 % of children were classified into the same or adjacent quintile, with the highest agreement for DHA (Table 6). In contrast, 3–7 % of children were misclassified into the opposite quintile. Kappa statistics (Table 6) showed that for the majority of *n*-6 and *n*-3 PUFA, there was poor agreement between their respective dietary and plasma measures (*κ* < 0·2). There was a moderate level of agreement between dietary and plasma DHA though (*κ* = 0·34, *P* < 0·001).

**Table 6.** Dietary PUFA intakes classified into quintiles, compared with quintiles of plasma PUFA proportions, with corresponding Cohen’s *κ* coefficients (*n* 4380) (Percentages)

Dietary^*^ and plasma PUFA	Same quintile (%)	Same or adjacent quintiles (%)	Opposite quintile (%)	Cohen’s Kappa (*κ*)		
				Cohen’s *κ* ^†^	95 % CI	*P*-value
Total *n*-6	22	56	6	0·122	0·09, 0·14	<0·001
18 : 2*n*-6 (LA)	24	58	6	0·155	0·13, 0·18	<0·001
20 : 4*n*-6 (AA)	23	56	7	0·079	0·05, 0·11	<0·001
Total *n*-3	23	59	5	0·185	0·16, 0·21	<0·001
18 : 3*n*-3 (ALA)	23	57	6	0·125	0·10, 0·15	<0·001
20 : 5*n*-3 (EPA)	22	54	6	0·096	0·07, 0·12	<0·001
22 : 6*n*-3 (DHA)	43	79	3	0·319	0·29, 0·35	<0·001

LA, linoleic acid; AA, arachidonic acid; ALA, *α*-linolenic acid.

* Dietary PUFA intakes are energy adjusted using the energy density method.

† Cohen’s Kappa analysis using weighted Kappa statistic (*κ*).

## Discussion

Dietary intakes of the *n*-6 and *n*-3 series of PUFA were assessed by FFQ in 7-year-old children living in South-West England in 1999–2000 and agreement with plasma PUFA measured 8 months later were assessed. On average, PUFA made up 6·5 % of TE intake, with the greatest proportion from *n*-6 PUFA (5·2 %) and only 0·7 % of energy from *n*-3 PUFA. This resulted in a *n*-6:*n*-3 ratio of 7·9:1. The majority of dietary *n*-6 PUFA were from fat spreads and cooking fat and from cereals and cereal-based products, whereas fish was the main source of LC-PUFA. In general, there were weak correlations between dietary PUFA and their corresponding plasma concentrations in blood. However, dietary DHA and plasma DHA concentrations had a moderate correlation and a reasonable level of agreement.

In this study, the intakes of *n*-6 and *n*-3 PUFA, as well as total fat, MUFA and SFA, were very similar to NDNS (1997) intake data on 4–10-year-old children^(19)^. The amount in g/d or percentage of energy from main PUFA subtypes (*n*-3, *n*-6, LA, AA, ALA, DHA and EPA) were also comparable with those reported in other studies of PUFA intakes in paediatric populations in Westernised countries^(13,14,15)^. However, the *n*-6:*n*-3 ratio (7·9:1) was generally lower than reported in these studies which could be due to the higher estimated *n*-3 PUFA intakes we observed (1·4 g/d compared with 0·88–1·3 g/d^(14,15,19)^). The low intakes of DHA and EPA observed in our study are also consistent with research in paediatric populations from other countries^(13,14,15,18)^.

The main food groups contributing to *n*-3 and *n*-6 PUFA intakes were very similar between the current study and the NDNS study of 4–10-year-olds^(19)^. However, we found that fat spreads and cooking oils, and cereal products contributed most to *n*-6 PUFA intake, while in the NDNS study, fats used in vegetable and potato dishes were the main source. As expected, fish and seafood dishes were the most important sources of LC-PUFA, contributing to 59 % of total DHA intake, which was comparable with previous findings^(13,14)^. According to the NDNS 2008–2012 rolling programme, white fish (including coated white fish) is the most common type of fish consumed in UK 6–11-year-olds (average intake is four times that of oily fish)^(17)^. Therefore, although white fish have much lower concentrations of EPA and DHA than oily fish because of its more frequent consumption, it formed the major source of LC-PUFA in these children (contributing to 51·9 % of EPA and 40·6 % DHA from total fish intake). Oily/fatty fish were an important source of dietary LC-PUFA though, consistent with findings from other studies in children^(13,16)^.

The mean daily intake of dietary PUFA in these 7-year-old children was in line with the Scientific Advisory Committee on Nutrition UK recommendation of 6·5 % of TE^(10,47)^. LA, the principal source of *n*-6, provided 5·1 % of TE in this cohort, also within the guidelines of ≥4 % of TE set by European Food Safety Authority^(12)^. In terms of *n*-3 PUFA dietary recommendations, the UK advocates that it forms a minimum of 0·2 % of food energy, while the FAO/WHO set an acceptable distribution range of 0·5–2·0 % of TE^(18)^. European Food Safety Authority recommends that ≥0·5 % of TE should come from the *n*-3 PUFA ALA. Therefore, the mean intakes of total *n*-3 PUFA (0·7 % of TE) and ALA (0·6 % of TE) in our study were within these dietary recommendations. However, the dietary intakes of the LC-PUFA in our study (85·4 mg/d) fell far below recommendations of 200–250 mg/d set by internationally recognised organisations^(12,18,47)^. In fact, none of the children in our cohort reached this level of intake and most children consumed less than half. This is not surprising considering the recent findings from the NDNS, which reported that only 4·7 % of UK children met the minimum recommendations for fish intake and only 4·5 % met minimum recommendations for oily fish^(17)^. Encouragingly, previous studies in children have shown that even eating a small amount of fish can significantly improve LC-PUFA levels compared with non-consumers^(48)^.

The ratio of *n*-6:*n*-3 PUFA in our study (7·9:1) is higher than what is considered for optimal growth and long-term health^(1)^, particularly cardiovascular health^(3,4,49)^. This ratio is a reflection of the abundance of food sources of LA in modern Western diets^(13,14)^, with regular use of fat spreads (margarines) and vegetable oils rich in LA (i.e. sunflower and maize oil) and their wide use in processed cereal-based products (baked goods and savoury and sweet snacks). In contrast, there are relatively fewer food sources high in *n*-3 PUFA. To improve the PUFA balance, a change in dietary habits is necessary, by increasing consumption of *n*-3 PUFA and/or decreasing consumption of *n*-6 PUFA. The advantage of decreasing *n*-6 PUFA intakes is that it potentiates the use of essential *n*-3 PUFA, since LA and AA compete for the same elongase and desaturase enzymes^(50)^. A higher intake of *n*-3 and LC-PUFA can be achieved by increasing the consumption of foods containing DHA and EPA (mainly fish and seafood) and/or foods containing their precursor, ALA. Although findings from the NDNS rolling programme comparing intake data from 1997 to 2008–2009 in 4–10-year-olds indicate that there was an overall shift towards recommended dietary guidelines for fat intakes, including an increase in consumption of *n*-3 PUFA, these related to relatively small increases in absolute terms^(19)^.

Our results showed weak-to-moderate correlations between dietary and plasma PUFA, consistent with results from previous studies comparing dietary PUFA intakes with tissue biomarkers in adults^(23,24,26,51)^ and paediatric populations^(27,28,29,30,52)^. A study of 0–11-year-olds from the USA compared FFQ estimates with the PUFA content of erythrocyte membranes and reported a correlation of 0·16 (*P* < 0·001) for *n*-6 PUFA, 0·25 (*P* = 0·001) for *n*-3 PUFA and 0·38 (*P* < 0·001) for total marine PUFA^(30)^, which is comparable to the correlations of these PUFA subtypes observed in our study. An Australian study in forty-seven healthy-weight children found moderate correlations between total dietary *n*-3 PUFA (*r* = 0·22) and EPA (*r* = 0·24) and their respective concentrations in erythrocyte membranes but no correlation with DHA^(27)^. Two studies in children observed higher correlations than in this study between dietary and tissue PUFA for total *n*-6 and LA (*r* ranging from 0·3 to 0·4)^(27,28)^. The different correlations reported between studies could be partly due to variations in the type of biomarker medium, dietary assessment method, period between obtaining dietary intake and biomarker tissue, health status of study population and genetic and lifestyle factors.

The overall weak-to-moderate correlations between dietary intakes of PUFA and their respective biomarkers observed in many studies, including ours, could be explained by the fact that tissue PUFA represent the interplay between dietary intakes, individual variation in absorption rates and metabolism. Metabolic processes and the complex interrelationships between different PUFA along the biosynthetic pathway of elongation and desaturation are a key reason why dietary intakes may not map directly onto plasma concentrations.

The weak correlations between eighteen-carbon chain PUFA intakes and plasma levels are in line with research in humans showing that the eighteen-carbon chain PUFA are largely oxidised^(53)^. An experimental study using tracers in rats supports this and found that in addition to oxidation, eighteen-chain PUFA move out of circulating blood lipids quickly and are stored in adipose tissue^(54)^. This could explain why blood eighteen-chain PUFA are not good indicators of dietary intake. In addition, the association between PUFA intakes and their biomarkers may differ for shorter- *v*. longer-chain PUFA. A systematic review of adult studies comparing FFQ estimated LC *n*-3 PUFA intake with plasma concentrations reported correlations in the range of 0·30–0·50 for DHA but only 0–0·28 for ALA^(22)^. Several studies in children have also found that correlations between PUFA in erythrocyte membranes, serum or plasma were generally higher for the marine-origin *n*-3 PUFA^(27,28,29,30)^. In our study, the correlations between the shorter-chain PUFA (LA and ALA) were generally weaker than the LC-PUFA. Shorter-chain PUFA may be less correlated with their tissue biomarkers because they are also converted into longer-chain PUFA, although this may only happen when concurrent intake of LC-PUFA is low^(55)^. ALA was not associated with plasma EPA and DHA in our study though, which is consistent with the poor endogenous conversion rate of ALA to DHA and EPA (with maximum conversion rates of 4 % and 8 %, respectively)^(3)^. Consequently, tissue and circulating LC-PUFA are mainly a reflection of their direct consumption from foods. This could explain why we observed a moderate correlation and level of agreement (according to Cohen’s *κ*) between dietary and plasma DHA. Indeed, in adult populations with high fish intakes, such as Japan, correlations of up to 0·60–0·70 for EPA and DHA have been observed^(56,57)^.

Our data showed some, although weak, evidence that dietary intakes of LA were associated with lower plasma concentrations of EPA. This is in line with the evidence indicating that higher concentration of LA inhibits the conversion of ALA to EPA. Inhibition occurs because the metabolic pathway involved in converting the PUFA precursors ALA and LA to their respective metabolites uses the same rate-limiting enzyme, delta-6 desaturase^(50)^. Intervention studies have also demonstrated that high intakes of LA were associated with lower conversion of ALA to EPA in subjects on diets without fish^(58,59)^.

The strengths and weaknesses of the study should be considered when interpreting these results. The strengths include the large number of children with both dietary and biomarker data, making this one of the largest correlation studies of this type in children. The majority of studies validating dietary assessment tools in children in the UK have a sample size of <50^(60)^. The use of a parental-completed FFQ specially designed for this age group enabled us to capture habitual dietary intakes, which is particularly advantageous when collecting information on foods such as fish and seafood, which are typically eaten less frequently in this population. We also had a complete database on quantities of EPA and DHA in the foods consumed, and data on intakes of these nutrients are limited in paediatric populations from the UK. However, we did not calculate intake of docosapentaenoic acid or its concentration in plasma: recent findings suggest that docosapentaenoic acid could be just as important as EPA and DHA in terms of health benefits linked to LC-PUFA^(61)^. Finally, the FFQ included five specific questions covering fish and seafood consumption that enabled us to assess the types of fish contributing to the LC-PUFA intake in these children.

In terms of study limitations, at birth, these children were relatively representative of the population in the area^(33)^. However, sample attrition during the 7-year follow-up is likely to have produced loss to follow-up bias, and it is probable that children with less healthy dietary patterns were under-represented which may in turn have influenced average PUFA intakes. However, the average PUFA intakes (as well as total fat, MUFA, SFA) and their main food sources reported in our study were very similar to the NDNS data on nationally representative UK 4–10-year-olds. Further attrition and subsequent bias occurred when obtaining a blood sample from these children as only 67·6 % of attendees at the research clinic agreed to this and these children had a lower BMI and energy intake and had a higher socio-economic status. Nevertheless, this should not have affected the correlation results, as these analyses were within subject. The use of parental-reported FFQ to assess children’s dietary intake would be subject to issues of reporting error and bias, as with all dietary survey methods to different extents^(60)^. To minimise this, the analysis excluded children with implausible dietary intakes.

A further limitation is that the FFQ, which was designed to assess habitual dietary intake, was completed approximately 8 months prior to the blood sample being obtained. However, plasma FA are an immediate biomarker which reflect intake over the past few days or meals^(62)^. The choice of medium for FA biomarker measurement is relevant because they reflect FA intakes over different time periods and so should ideally be time integrated with the dietary intake period being measured^(21)^. Erythrocyte membranes reflect intake aggregated over approximately 4 months. However, two studies in paediatric populations that compared FFQ-estimated PUFA intakes with PUFA levels in erythrocyte membranes reported similar ranges of correlation coefficient to our study^(27,30)^. In addition, eating habits have been shown to be reasonably stable during childhood, with moderate tracking levels^(63)^. NDNS data on the time trends in *n*-6 and *n*-3 FA in UK 7–9-year-old children show that there are minimal differences in intakes over this period in childhood^(64)^.

The difference in reference period between the FFQ and biomarker assessment could mean that the observed correlations were an underestimation of the true correlations^(26)^. The storage time of the samples is also a potential limitation, due to oxidation of PUFA and deterioration of lipid classes over time^(65)^. In our study, the samples were stored at −70°C for approximately 10 years before the plasma FA composition was analysed. However, plasma FA are considered to be relatively stable for up to 10 years with such ultracold storage^(64,65)^. This also supports our choice of pool sample (plasma) in place of erythrocytes; although erythrocytes are less influenced by recent dietary intake, the FA composition is not completely stable during their 4-month lifespan, since the FA in the membranes can remodel with recent diet intake and the haem content of erythrocytes can cause PUFA oxidation^(65)^.

A final limitation is the food composition database used to estimate intakes from the FFQ data. The composition of foods and types of food available change with time (e.g. *n*-3-enriched foods are now more readily available). Food composition databases are limited in both the number of foods they contain and the frequency that they update food composition data. However, we supplemented the M&W food composition tables with up-to-date data from other sources in order to maximise the completeness of the PUFA composition of the foods covered in our FFQ.

In conclusion, the weak to moderate correlations between dietary and plasma LC-PUFA intakes and good level of agreement in cross-classiﬁcation analysis reflect the ability of the parental-completed FFQ to relatively rank the LC-PUFA intakes in this paediatric population, particularly for DHA. Our results highlight the need for public health initiatives to address the suboptimal ratio of *n*-6:*n*-3 PUFA and very low *n*-3 LC-PUFA in school-age children in the UK. The optimal dietary approach to increase tissue LC-PUFA and to reach recommended intakes is to consume them directly in their preformed state, mainly from sustainably sourced fish (particularly oily fish) and seafood, but also from lean (red) meat, eggs and products nutritionally enriched with LC-PUFA. For children unable or reluctant to eat fish or seafood, then dietary changes that reduce foods high in LA (i.e. sunflower and maize oil and cereal-based processed products) while increasing foods rich in ALA (i.e. rapeseed and flaxseed oil, nuts, green leafy vegetables and whole wheat bread) can improve their *n*-3 FA status.

## Supplementary material

For supplementary material accompanying this paper visit https://doi.org/10.1017/S0007114521002191. click here to view supplementary material
