# Supplementary material for: The relationship between dietary intakes and plasma concentrations of PUFA in school-age children from the Avon Longitudinal Study of Parents and Children (ALSPAC) cohort
Source: Br J Nutr. 2021 Jun 17;127(9):1367–77. doi: 10.1017/S0007114521002191 (PMC10484628; doi:10.1017/S0007114521002191)
Supplement: Supplementary file 1 [file S0007114521002191sup.zip › S0007114521002191sup001.docx]

**Supplementary Material**

# The relationship between dietary intakes and plasma concentrations of polyunsaturated fatty acids in school-aged children from the ALSPAC cohort.

G Buckland, S de Silva Johnson, L Johnson, C Taylor_,_ LR Jones, PM Emmett.

**Supplementary Table 1.** Daily dietary intakes of PUFAs by sex, estimated from a FFQ in 7-year old children from ALSPAC.

**Supplementary Table 2.** Plasma fatty acids proportions by sex in 7-year old children

from ALSPAC (n=4,380).

|  |  |  |  |  |
| --- | --- | --- | --- | --- |
| Plasma proportion as % of total fatty acids | Female |  | Male | P-value Difference between sexes^1^ |
|  | Median (IQR) |  | Median (IQR) |  |
| Saturated fatty acids | 29.6 (27.3-31.4) |  | 29.7 (27.5-31.5) | 0.022 |
| Monounsaturated fatty acids | 26.9 (25.1-29.0) |  | 26.4 (24.5-28.5) | <0.001 |
| n-6 Polyunsaturated fatty acids | 39.6 (37.2-42.2) |  | 40.1 (37.4-42.5) | 0.011 |
| 18:2 n-6 (LA) | 30.6 (28.6-32.7) |  | 30.7 (28.6-32.7) | 0.859 |
| 20:4 n-6 (AA) | 6.3 (5.5-7.2) |  | 6.4 (5.5-7.4) | <0.001 |
| n-3 Polyunsaturated fatty acids | 3.8 (3.4-4.3) |  | 3.8 (3.3-4.3) | 0.350 |
| 18:3 n-3 (ALA) | 0.7 (0.5-0.8) |  | 0.6 (0.5-0.8) | 0.007 |
| 22:6 n-3 (DHA) | 1.8 (1.6-2.2) |  | 1.8 (1.5-2.2) | 0.006 |
| 20:5 n-3 (EPA) | 0.6 (0.5-0.7) |  | 0.6 (0.5-0.8) | 0.167 |
| Abbreviations; SD:Standard Deviation. IQR: Inter Quartile Range (25th percentile-75th percentile). n-6: omega-6 series. n-3: omega-3 series. LA: Linolenic acid. AA: Arachidonic acid. ALA: Alpha-linolenic acid. DHA: Docosahexaenoic acid. EPA: Eicosapentaenoic acid. ^1^Wilcoxon Man-Whitney test | | | | |

**Supplementary Table 3**. Daily intake and percentage contribution of total n-6 and total n-3 PUFA intakes by food group and by sex, estimated from a parental-completed food frequency questionnaire when the child was aged 7 years (n=8,242).

**Supplementary Table 4.** Contribution of different types of fish to DHA and EPA intake by sex, estimated from a parental-completed food frequency questionnaire when the child was aged 7 years (n=8,242).

**Supplementary Table 5a.** Spearman's Correlation Coefficients (r) between plasma concentrations and energy adjusted dietary intakes of n-3 and n-6 PUFAs in females (n=2,114).

**Supplementary Table 5b** Spearman's Correlation Coefficients (r) between plasma concentrations and energy adjusted dietary intakes of n-3 and n-6 PUFAs in males (n=2,266).
